# Decreased Serum Uric Acid Level as an Indicator of Altered Oxidative Balance in Patients With Migraine

**DOI:** 10.7759/cureus.32136

**Published:** 2022-12-02

**Authors:** Sedat Yasin, Erman Altunisik

**Affiliations:** 1 Neurology, Gaziantep University Faculty of Medicine, Adiyaman, TUR; 2 Neurology, Adiyaman University School of Medicine, Adiyaman, TUR

**Keywords:** albumin, chronic migraine, uric acid, migraine, oxidative stress

## Abstract

Introduction: Migraine is one of the most common neurological diseases. The pathophysiology of migraine has not yet been fully elucidated. There is increasing evidence supporting the relationship between oxidative stress and migraine.

Method: This is a retrospective, cross-sectional and observational study. The patients were divided into two groups, episodic migraine and chronic migraine. Episodic migraine patients were divided into two subgroups, migraine with aura and migraine without aura. Serum Albumin, total bilirubin, uric acid levels, and migraine clinical findings were obtained from medical records.

Results: A total of 181 participants, 88 patients and 93 controls, were included in the study. Serum albumin levels were lower in the patient group than in the control group, they did not reach statistical significance (p=0.082). There was no significant difference between the patient and control groups for total bilirubin levels (p=0.785). Serum uric acid levels in the patient group were found to be significantly lower than in the control group (p<0.001). Measured levels were similar in chronic and episodic migraine groups, and migraine with aura and migraine without aura subgroups.

Conclusion: We thought this oxidative stress marker may be associated with the presence of migraine, but this is not significant for migraine subtypes and migraine progression.

## Introduction

Migraine is a chronic neurological disease characterized by throbbing headache attacks, usually unilateral, aggravated by physical activity, accompanied by photophobia, phonophobia, nausea, and vomiting [1]. It has a prevalence of 18% in women and 6% in men over a one-year period, peaking between the ages of 25 and 55. Migraine attacks can significantly impair functional ability in work, school, home, and social settings [2]. Chronic migraine (CM) is considered a headache lasting more than 15 days per month, with a migraine attack lasting at least eight days for more than three months. The presence of headaches less than 15 days per month (<8 days of a migraine attack) is called episodic migraine (EM) [3].

The pathophysiology of migraine has not yet been fully elucidated. Different mechanisms have been proposed to date including vascular dysfunction, neurogenic inflammation, and activation of the trigeminovascular pathway [4]. There is increasing evidence supporting the relationship between neurogenic inflammation and migraine [5]. Studies have reported that decreased antioxidant levels, which disrupt the oxidative balance in migraine patients, may trigger a migraine attack by causing neurogenic inflammation [6]. Migraine aura, which is thought to be associated with cortical spreading depression, is also thought to be a response to oxidative stress [7]. Although it has been hypothesized that oxidative stress plays an important role in the pathogenesis of migraine, there are relatively few studies on blood markers of antioxidant status in migraine.

Serum albumin (ALB), total bilirubin (TBIL), and uric acid (UA) are major non-enzymatic antioxidants in human plasma and may play important roles in scavenging endogenous reactive oxygen species (ROS) [8]. UA is the end product of purine metabolism [9]. UA is an effective extracellular free radical scavenger and is known to be responsible for 25% of the total antioxidant capacity of plasma [10]. UA is an endogenous antioxidant that inhibits inflammatory responses, reduces the permeability of the blood-brain barrier (BBB), and has neuroprotective effects [11]. Low serum UA levels have been identified as a risk factor for neurodegenerative diseases [12].

Studies measuring serum levels of antioxidants in migraine are limited. In this study, it was aimed to investigate the effects of major non-enzymatic antioxidants such as ALB, TBIL, and UA in the serum on the pathogenesis and chronicity of the disease by measuring the levels in migraine patients.

## Materials and methods

In this retrospective, cross-sectional and observational study, a total of 88 patients and 93 healthy control groups included, aged between 18-60 years, admitted to the neurology headache outpatient clinic of a tertiary university hospital between 01 January 2018 and 01 January 2022 and were diagnosed with migraine according to the International Classification of Headache Disorders (ICHD)-3 beta criteria [1]. The patients were divided into two groups EM and CM. EM patients were divided into two subgroups migraine with aura (MwA) and migraine without aura (MwoA). For each patient, the characteristic clinical findings of migraine, such as disease duration, frequency of migraine attacks, duration of attacks, number of headache days per month, presence of aura, migraine severity, and sociodemographic characteristics were obtained from medical records. Migraine severity was evaluated by a neurologist specializing in headaches according to the Migraine Disability Assessment Score (MIDAS) [13].

Those with chronic systemic disease (such as chronic kidney failure, chronic liver disease), malignancy, acute or chronic infection, endocrine-metabolic disease, rheumatic-immunological disease, and hematological disease (such as polycythemia, anemia), those who use cigarette alcohol, those who use topiramate and diuretic drugs, patients with gastrointestinal insufficiency and malnutrition, and patients whose medical records could not obtain serum ALB, TBIL and UA values and migraine clinical findings were not included in the study. In addition to the selection criteria used for the patient group, the control group consisted of healthy individuals who did not have a history of any type of headache and gave blood samples for routine health screening. Blood samples were taken from the antecubital vein from the patient in the attack-free period and control groups after 8-10 hours of fasting and sent to the hospital laboratory.

The study was conducted in accordance with the Declaration of Helsinki guidelines and approval was obtained from the ethics committee of Gaziantep University Faculty of Medicine (approval number 2022/427).

Statistical analysis

Statistical analyses were performed with the help of SPSS Statistics, version 22.0 (IBM Corp., Armonk, NY). Mean and standard deviation values were used for descriptive analyses. The conformity of the quantitative variables to the normal distribution was analyzed by kurtosis and skewness tests. Categorical variables were compared with the Chi-square Test. The independent T-test was used to compare the quantitative variables with normal distribution, and the Mann Whitney U Test was used to compare the quantitative variables that did not fit the normal distribution. Comparisons between categorical variables with more than two groups were made with one-way ANOVA and Kruskal Wallis tests. The inter-correlation of quantitative data was analyzed with Spearman’s and Pearson’s correlation tests. A p-value below 0.05 was considered statistically significant.

## Results

A total of 181 participants, 88 patients and 93 controls, were included in the study. While 55 migraineurs were in the EM group, 33 were in the CM group. Twelve of the EM patients were in the MwA subgroup and 43 were in the MwoA subgroup. There were 72 (81.8%) women in the patient group and 76 (81.7%) in the control group. The mean age of the patient group was 35.28, and the mean age of the control group was 35.15. The patient and control groups are similar in terms of age and gender distribution. Serum ALB, TBIL, and UA values were 4.16 g/dl, 0.50 mg/dl, 3.91 mg/dl in the patient group, and 4.26 g/dl, 0.53 mg/dl, and 4.90 mg/dl in the control group, respectively. Although serum ALB values were lower in the patient group than in the control group, they did not reach statistical significance (p=0.082). There was no significant difference between the patient and control groups for TBIL values (p=0.785). Serum UA values in the patient group were found to be significantly lower than in the control group (p<0.001). Demographic characteristics and laboratory data of the patient and control groups are shown in Table 1.

**Table 1:** Clinical features and laboratory data of patients with migraine and controls. BMI, body mass index; ALB, albümin; TBIL, total bilirubin; UA, uric acid

	Migraine (n=88)	Control (n=93)	p
Age (year) mean±Sd	35.28±9.92	35.15±7.98	0.920
Gender (male/female)	16/72	17/76	0.986
BMI (kg/m^2^) mean±Sd	24.13±2.16	23.9±2.08	0.812
ALB (g/dL) mean±Sd	4.16±0.39	4.26±0.36	0.082
TBIL (mg/dL) mean±Sd	0.50±0.01	0.53±0.02	0.785
UA (mg/dl) mean±Sd	3.91±0.96	4.90±1.08	<0.001

There was no significant difference between the EM and CM groups for serum ALB, TBIL, and UA values (p 0.333, 0.795, 0.323, respectively). Migraine clinical findings and laboratory data of EM and CM patients are shown in Table 2.

**Table 2:** Comparison of headache characteristics and laboratory data between chronic and episodic patients ALB, albümin; TBIL, total bilirubin; UA, uric acid; MIDAS, Migraine Disability Assessment Score

	Episodic Migraine (n=55) mean±Sd	Chronic migraine (n=33) mean±Sd	p
Disease duration (year)	8.21±7.03	9.30±5.90	0.460
Frequency of attacks per month	3.58±1.67	9.06±2.63	<0.001
Attack duration (hour)	43.20±20.67	59.87±19.73	<0.001
Number of headache days per month	7.70±3.83	19.84±3.95	<0.001
Headache severity (MIDAS score)	11.52±0.94	33.30±3.25	<0.001
ALB (g/dL)	4.11±0.39	4.23±0.38	0.333
TBIL (mg/dL)	0.51±0.02	0.47±0.02	0.795
UA (mg/dl)	3.82±0.94	4.06±0.99	0.323

In the EM subgroup, no significant difference was found between MwA and MwoA for serum ALB, TBIL, and UA values (p 0.600, 0.202, 0.474, respectively). Laboratory data of MwA and MwoA patients are shown in Table 3. In the correlation analysis, no significant correlation was found between serum ALB, TBIL, and UA values and disease duration, frequency of migraine attacks, duration of attacks, number of headache days per month, and MIDAS (Migraine Disability Assessment) scores.

**Table 3:** Comparison of laboratory data between Migraine with aura and Migraine without aura patients ALB, albümin; TBIL, total bilirubin; UA, uric acid

	Migraine with aura (n=12) mean±Sd	Migraine without aura (n=43) mean±Sd	p
ALB (g/dL)	4.06±0.43	4.13±0.38	0.600
TBIL (mg/dL)	0.59±0.06	0.49±0.03	0.407
UA (mg/dl)	3.65±0.98	3.87±0.94	0.474

## Discussion

Oxidative stress occurs as a result of insufficient antioxidants in the elimination of ROS and causes various diseases if not controlled [14]. Neurons are susceptible to exogenous and endogenous damage mediated by ROS. Oxidative stress is blamed for many neurological disorders, especially migraine [15]. In this study, we found that serum levels of UA, an effective extracellular free radical scavenger, were decreased in migraine patients.

The precise mechanisms by which oxidative stress may play a role in the pathophysiology of headaches have not been clearly defined. It is hypothesized that the increased production of ROS may lead to peroxidation of cell membrane phospholipids, destruction of intracellular molecules (especially proteins and DNA), and consequently deterioration in the normal function of cells [16]. In particular, the inflammatory response resulting from oxidative stress is thought to play an important role in the pathogenesis of acute migraine-related pain [16,17]. It was determined many years ago that the levels of superoxide dismutase enzyme, which is one of the important components of the antioxidant mechanism, are low in migraine patients [18]. In a systematic meta-analysis, it was reported that the oxidative stress created by the nitric oxide pathway is high in migraine patients [19]. In a different study, serum ALB, TBIL, and UA levels were found to be significantly lower in migraine patients compared to the control group [20]. In another study, serum UA levels were found to be significantly lower in migraine patients [21]. In another study, while serum UA values were found to be similar in migraine and control groups, it was observed that UA values in the migraine attack period were significantly lower than in the attack-free period [22]. In our study, serum UA values were found to be low in migraine patients, in line with previous studies. Low UA levels have been defined as a risk factor for some chronic neurological diseases [23,24]. In a study, it was reported that the incidence of migraine was low in gout with high uric acid levels [22]. The decrease in this antioxidant, which is thought to suppress the inflammatory response and stabilize the BBB (blood-brain barrier), may have contributed to the triggering of migraine attacks by neuronal hyperexcitability and activation of the trigeminovascular pathway by disrupting the oxidative balance.

It has been thought that oxidative stress and insufficient antioxidant defense may play a role in the chronicity of migraine [15]. Previous studies have shown that peripheral inflammation plays a role in disease progression in migraine [25]. Cytokines induced by oxidative stress may increase pain sensitivity by acting on peripheral nociceptive nerve terminals and sensory ganglia [26]. In a different study, it was shown that as the frequency of headache attacks increased, the level of oxidative stress biomarkers increased and antioxidant defense decreased [27]. In our study, serum ALB, TBIL, and UA levels were found to be similar in the EM and CM groups. It can be thought that the change in oxidative balance does not differ according to migraine subgroups and affects all migraine groups to a similar extent. We think that multifactorial causes such as psychosocial, genetic and environmental factors may be more effective in the progression of migraine to the chronic phase.

Determination of oxidative changes in patients with migraine may contribute to the determination of treatment planning and treatment success in addition to its diagnostic contributions. Approaches that increase antioxidant capacity such as agents with antioxidant content and lifestyle changes such as dietary supplements, regular physical exercise and elimination of psychosocial stressors may be promising in migraine prophylaxis. To date, possible antioxidant treatment options such as dietary supplements containing riboflavin, coenzyme Q10, melatonin, magnesium, vitamin D, and polyunsaturated fatty acids have been recommended in migraine [28,29].

Our study has some limitations. First of all, our retrospective, cross-sectional and observational study does not provide conclusive evidence that oxidative stress plays a role in the pathogenesis of migraine. The results of our study, which included a small patient population from a single center, cannot be generalized. Differences such as diet, physical activity level, and psychosocial factors may have affected the results of the study. In addition, valuable parameters such as total antioxidant levels, total oxidant levels, and oxidative stress index were not measured.

## Conclusions

In our study, we found that serum UA levels were low in migraine patients. We thought that this oxidative stress marker may be associated with the presence of migraine, but this is not significant for migraine subtypes and migraine chronicity. Studies focusing on oxidative balance may be valuable in understanding the underlying mechanism of migraine. In addition, investigating the effects of the agents used in the treatment of migraine on oxidative stress biomarkers may further elucidate the pathogenesis of the disease. We think that prospective cohort studies with a large population will be useful.

## References

[1] (2018). Headache Classification Committee of the International Headache Society (IHS) The International Classification of Headache Disorders, 3rd edition. Cephalalgia.

[2] Buse DC, Scher AI, Dodick DW, Reed ML, Fanning KM, Manack Adams A, Lipton RB (2016). Impact of migraine on the family: perspectives of people with migraine and their spouse/domestic partner in the CaMEO study. Mayo Clin Proc.

[3] Buse DC, Greisman JD, Baigi K, Lipton RB (2019). Migraine progression: a systematic review. Headache.

[4] Dodick DW (2018). A phase-by-phase review of migraine pathophysiology. Headache.

[5] Erdener ŞE, Kaya Z, Dalkara T (2021). Parenchymal neuroinflammatory signaling and dural neurogenic inflammation in migraine. J Headache Pain.

[6] Yazar HO, Yazar T, Aygün A, Kaygisiz Ş, Kirbaş D (2020). Evaluation of simple inflammatory blood parameters in patients with migraine. Ir J Med Sci.

[7] Shatillo A, Koroleva K, Giniatullina R (2013). Cortical spreading depression induces oxidative stress in the trigeminal nociceptive system. Neuroscience.

[8] Xie KH, Liu LL, Su CY (2020). Low antioxidant status of serum uric acid, bilirubin, albumin, and creatinine in patients with benign paroxysmal positional vertigo. Front Neurol.

[9] Shaw H, Konidena A, Malhotra A, Yumnam N, Farooq F, Bansal V (2020). Psychological status and uric acid levels in oral lichen planus patients - a case-control study. Indian J Dent Res.

[10] Basaga HS (1990). Biochemical aspects of free radicals. Biochem Cell Biol.

[11] Yazar HO, Yazar T, Yildirim T, Keşkek A, Altunkaynak Y (2019). Assessment of serum uric acid levels in patients with restless legs syndrome. Acta Neurol Belg.

[12] Brody DM, Litvan I, Warner S (2016). Relationship between uric acid levels and progressive supranuclear palsy. Mov Disord.

[13] Stewart WF, Lipton RB, Dowson AJ, Sawyer J (2001). Development and testing of the Migraine Disability Assessment (MIDAS) Questionnaire to assess headache-related disability. Neurology.

[14] Yigit M, Sogut O, Tataroglu Ö (2018). Oxidative/antioxidative status, lymphocyte DNA damage, and urotensin-2 receptor level in patients with migraine attacks. Neuropsychiatr Dis Treat.

[15] Naduthota RM, Bharath RD, Jhunjhunwala K, Yadav R, Saini J, Christopher R, Pal PK (2017). Imaging biomarker correlates with oxidative stress in Parkinson's disease. Neurol India.

[16] Eren Y, Dirik E, Neşelioğlu S, Erel Ö (2015). Oxidative stress and decreased thiol level in patients with migraine: cross-sectional study. Acta Neurol Belg.

[17] Geyik S, Altunısık E, Neyal AM, Taysi S (2016). Oxidative stress and DNA damage in patients with migraine. J Headache Pain.

[18] Shimomura T, Kowa H, Nakano T, Kitano A, Marukawa H, Urakami K, Takahashi K (1994). Platelet superoxide dismutase in migraine and tension-type headache. Cephalalgia.

[19] Neri M, Frustaci A, Milic M, Valdiglesias V, Fini M, Bonassi S, Barbanti P (2015). A meta-analysis of biomarkers related to oxidative stress and nitric oxide pathway in migraine. Cephalalgia.

[20] Yang Z, Xu P, Geng C, Zhang H (2022). Evaluation of simple antioxidant blood parameters in patients with migraine. Front Neurol.

[21] Yazar T, Yazar HO, Aygün A, Karabacak V, Altunkaynak Y, Kirbaş D (2021). Evaluation of serum uric levels in migraine. Neurol Sci.

[22] Erdal N, Altunkaynak Y, Altunkaynak E, Öztürk M, Mutluay B, Köksal A, Baybaş S (2005). Evaluation of lipid peroxidation representing oxidative stress in patients with migraine (Article in Turkish). Düşünen Adam.

[23] Chiurchiù V, Orlacchio A, Maccarrone M (2016). Is modulation of oxidative stress an answer? The state of the art of redox therapeutic actions in neurodegenerative diseases. Oxid Med Cell Longev.

[24] Vieru E, Köksal A, Mutluay B, Dirican AC, Altunkaynak Y, Baybas S (2016). The relation of serum uric acid levels with L-Dopa treatment and progression in patients with Parkinson's disease. Neurol Sci.

[25] Taheri M, Nicknafs F, Hesami O, Javadi A, Arsang-Jang S, Sayad A, Ghafouri-Fard S (2021). Differential expression of cytokine-coding genes among migraine patients with and without aura and normal subjects. J Mol Neurosci.

[26] Aczél T, Körtési T, Kun J (2021). Identification of disease- and headache-specific mediators and pathways in migraine using blood transcriptomic and metabolomic analysis. J Headache Pain.

[27] Togha M, Razeghi Jahromi S, Ghorbani Z, Ghaemi A, Rafiee P (2019). An investigation of oxidant/antioxidant balance in patients with migraine: a case-control study. BMC Neurol.

[28] Ghorbani Z, Togha M, Rafiee P (2019). Vitamin D in migraine headache: a comprehensive review on literature. Neurol Sci.

[29] Nattagh-Eshtivani E, Sani MA, Dahri M, Ghalichi F, Ghavami A, Arjang P, Tarighat-Esfanjani A (2018). The role of nutrients in the pathogenesis and treatment of migraine headaches: review. Biomed Pharmacother.

